# Curcumin Protects Intestinal Mucosal Barrier Function of Rat Enteritis via Activation of MKP-1 and Attenuation of p38 and NF-κB Activation

**DOI:** 10.1371/journal.pone.0012969

**Published:** 2010-09-24

**Authors:** Wei-Bing Song, Yuan-Yuan Wang, Fan-Su Meng, Qing-Hua Zhang, Jian-Ying Zeng, Li-Ping Xiao, Xin-Pei Yu, Dan-dan Peng, Lei Su, Bing Xiao, Zhen-Shu Zhang

**Affiliations:** 1 Department of Gerontology, General Hospital of Guangzhou Military Command of People's Liberation Army, Guangzhou, China; 2 Guangdong Provincial Key Laboratory of Gastroenterology, Department of Gastroenterology, Nanfang Hospital, Southern Medical University, Guangzhou, China; 3 Department of Intensive Care Unit, Traditional Chinese Medical Hospital of ZhongSan City, Zhongshan, China; 4 Department of Gerontology, 458 Hospital of People's Liberation Army, Guangzhou, China; Charité-Universitätsmedizin Berlin, Germany

## Abstract

**Background:**

Intestinal mucosa barrier (IMB) dysfunction results in many notorious diseases for which there are currently few effective treatments. We studied curcumin's protective effect on IMB and examined its mechanism by using methotrexate (MTX) induced rat enteritis model and lipopolysaccharide (LPS) treated cell death model.

**Methodology/Principal Findings:**

Curcumin was intragastrically administrated from the first day, models were made for 7 days. Cells were treated with curcumin for 30 min before exposure to LPS. Rat intestinal mucosa was collected for evaluation of pathological changes. We detected the activities of D-lactate and diamine oxidase (DAO) according to previous research and measured the levels of myeloperoxidase (MPO) and superoxide dismutase (SOD) by colorimetric method. Intercellular adhesion molecule-1 (ICAM-1), tumor necrosis factor α (TNF-α) and interleukin 1β (IL-1β) were determined by RT-PCR and IL-10 production was determined by ELISA. We found Curcumin decreased the levels of D-lactate, DAO, MPO, ICAM-1, IL-1β and TNF-α, but increased the levels of IL-10 and SOD in rat models. We further confirmed mitogen-activated protein kinase phosphatase-1 (MKP-1) was activated but phospho-p38 was inhibited by curcumin by western blot assay. Finally, NF-κB translocation was monitored by immunofluorescent staining. We showed that curcumin repressed I-κB and interfered with the translocation of NF-κB into nucleus.

**Conclusions/Significance:**

The effect of curcumin is mediated by the MKP-1-dependent inactivation of p38 and inhibition of NF-κB-mediated transcription. Curcumin, with anti-inflammatory and anti-oxidant activities may be used as an effective reagent for protecting intestinal mucosa barrier and other related intestinal diseases.

## Introduction

Intestinal mucosa barrier (IMB), the first line of defense against hostile environment, is composed of a single layer of columnar epithelium and inter-epithelial tight junctions. It has the function of selectively absorption nutrients and resists pathogens, toxins and inflammatory factors invasion. Clinical and lab researches showed that variety of factors could weaken or undermine the intestinal barrier structure and function, such as trauma, sepsis[1], operation, chemotherapy, radiotherapy, severe pancreatitis, long-term parenteral nutrition, and so on, thus resulted in bacteria translocation, subsequently led to endogenous infection and endotoxiemia (ETM). The final consequence of IMBdysfunction is systemic inflammation response syndrome (SIRS) and multiple organs dysfunction syndrome (MODS) [2]. Intestine is considered to be not only the target organ of MODS, but also the initiator of MODS. Therefore, IMB function has become an important prognostic indicator for critically ill patients [3].

Correctly assessing IMB function is very important for evaluating the patient's condition, estimating prognosis, and providing comprehensive treatment. However, due to no difficulty for directly observation of intestinal barrier function, currently work is mostly carried out byexamining indirect molecular level. D-lactate, a particular final metabolic product of bacteria in gastrointestinal tract, will release into blood when intestinal mucosa barrier is damaged. Examination of D-lactate in peripheral blood can evaluate damage situation of intestinal mucosa because of lack of D-lactate dehydrogenase in mammals [4]. The other indicator is Diamine oxidase (DAO), one kind of endocellular enzyme, only exists in villus cytoplasm of intestinal stratum supravasculare in mammals. When intestinal epithelial cells are injured, endocellular DAO released into the intestinal intercellular space, entered into the lymph vessel and the blood, finally resulted a stable high level of DAO in blood plasma. Hence, the activity of DAO in blood indicates maturity and integrity of intestinal mucosa [5]. Above all, the evaluation of IMB function is depended on these two indicators in our research.

Based on an accurate assessment of IMB function, here, we intend to find a proper drug capable for protecting it, and thus for prevention and treatment of intestinal inflammation [6]–[8]. In recent years, researchers focused on the traditional Chinese medicine for its better therapeutic effects and less toxic side effects. Curcumin,isolated from the rhizomes of the plant Curcuma longa Linn, has anti-inflammation, anti-oxidization and free radical removal effects [9]. Tremendous research papers have reported intriguing pharmacologic effects associated with curcumin. It can attenuate experimental colitis by inhibiting the activation of NF-κB and reducing the activity of p38 MAPK[10]–[12]. Curcumin can also suppress the activation of NF-κB in ethanol-induced liver injury in rats [13]. The previous research showed that the inhibition of inhibitory factor I-κB kinase activity is a possible mechanism by which curcumin blocks NF-κB activation [14]. Moreover, curcumin was proved to suppress the p38, JNK and NF-κB p65 in human intestinal epithelial HT29 cell line. Curcumin also attenuated Stx-1 induced cell death [15]. Hence, the evidences from the studies both *in vitro* and *in vivo* indicated that curcumin acted as a protective reagent against inflammation or infection.

Unfortunately, how the mechanism of curcumin mediates the effect mentioned above is still unknown. In this study, using rat model of enteritis and intestinal epithelial damage, we evaluated the protective role of curcumin on IMB function. Then, we establishedthe model of cell damage to identify the potentially activation of epithelial intra- and extra-cellular MAKP and NF-κB signaling pathways. These data clarified the molecular mechanism of curcumin in protecting IMB. Our study provides a traditional Chinese medicine, curcumin, for treating IMB dysfunction and improving inflammatory bowel diseases (IBD) and other related intestinal diseases.

## Methods

### 1 Ethics Statement

All experimental procedures on rats were approved by the Committee on the Ethics of Animal Experiments of Southern Medical University (Permit Number: 14-2527). All surgery was performed under sodium pentobarbital anesthesia, and all efforts were made to minimize suffering.

### 2 Animals and blood samples

SD rats, weighing 200–250 g (10–12weeks), purchased from the Laboratory Animal Center (Southern Medical University, China) were used. Rats were raised under standard conditions (12-hour day-night rhythm) in the Animal Care Facility Service (Southern Medical University, China). This study was carried out in strict accordance with the recommendations in the Guide for the Care and Use of Laboratory Animals of the National Institutes of Health. 15 rats were used as minimum sample size per group in all animal experiments.

### 3 MTX-induced enteritis rat model

**Figure 1:**
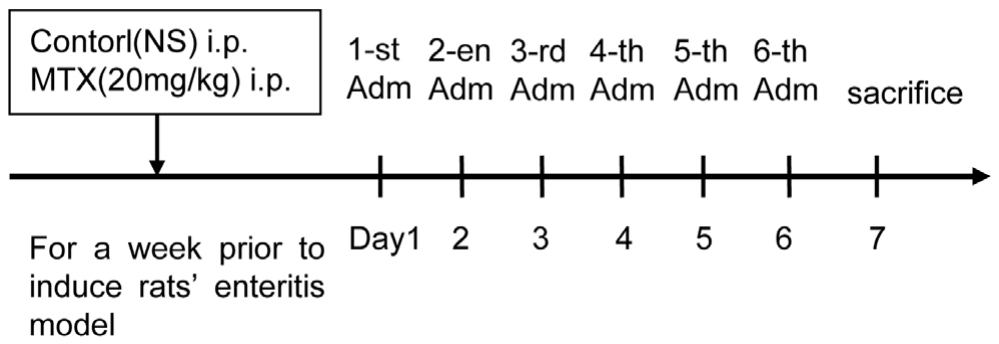
Construction of MTX induced enteritis animal model. Schedule of rat experimental procedure is shown by a diagram. Enteritis was induced in rats through peritoneal injection of MTX (20 mg/kg). Control group was only treated with saline. From the first day that the rat models were made, the drugs, curcumin or NAC, were intragastrically administrated with the specified dosage once a day for 7 days, then all rats were anesthetized and killed.

Enteritis was induced in rats through peritoneal injection of MTX (20 mg/kg) [16]. Rats were randomly divided into 4 groups: control group (peritoneal injection of normal saline only), MTX group (MTX, 20 mg/kg), MTX+curcumin group (MTX, 20 mg/kg; curcumin, 100 mg/kg) and MTX+NAC positive control group (MTX, 20 mg/kg; NAC, 150 mg/kg). From the first day that the rat models were made, different drugs were intragastrically administrated with the specified dosage once a day for 7 days. Rats in control group and MTX group were injected with saline (Fig. 1).

### 4 Reagents and apparatus

Curcumin (2 g/L, 95%), D-lactate standard solution and D-lactate dehydrogenase, O-dianisidine, cadaverine dihydrochloride, horseradish peroxidase, LPS, bovine insulin, DAO standard solution and SB203580 (SB, inhibitor of p38) were all purchased from Sigma company. N-acetylcysteine (NAC) and MTX were purchased in Zhejiang Wanma Pharmaceutical Co., Ltd. and Shanghai Sunve Pharmaceutical Co., Ltd. respectively.

### 5 The disease activity index (DAI), colonic mucosal damage index (CMDI) and histological score (HS) of the rats were evaluated

DAI were evaluated based on the general symptoms of rats during the disease progress including weight decent percentage, stool viscidity and bloody stools according to the scoring system [17]. On the 7th day animal models were made, the rats were killed, and the intestinal segments of rats were observed with unaided eye to determine the CMDI [18]. Severity of colitis was graded on a scale of 0–IV and defined as the pathological index according to the standard scoring system [19]. Finally, the intestinal mucosa samples were fixed in 10% formaldehyde solution at room temperature according to standard method. Briefly, samples were embedded in paraffin, then sectioned at 5 µm, and stained with Hematoxylin-Eosin, picked up on glass slides for light microscopy. All samples were evaluated and by an experienced pathologist who is blinded to the experiment. Finally, we got histological score (HS) for each samples [20].

### 6 Rats blood samples

7 days after MTX injection, the rats were anesthetized and a 3 mL sample of venous blood was collected. The blood samples were injected into dry test tubes and separated by centrifugation, the serum was stored at −20°C until use.

### 7 Detection of the levels of plasma D-lactate and DAO in the small intestinal mucosa by spectrophotometry

After the plasma was deproteined with perchloric acid, the levels of D-lactate and DAO in the serum were detected by spectrophotometry as the same method described in reference lectures [12], [17].

### 8 Detection of MPO and SOD Activity in intestinal mucosa

7 days after MTX injection, the rats were anesthetized, the abdomens were opened along the median line, and the small intestine was rapidly excised, the rinsed gently with ice-cold PBS, placed on ice, and opened longitudinally. The small intestine was incised, and the fecal contents were washed out gently with 2−3 ml of PBS. The ileum within 0.5 cm of the ileocecal junction (about 1 cm) was excised with a sharp scalpel, weighted, fixed with 3 fold phosphate buffer (0.1 M, pH 7.2), homogenated, and centrifugalized (10000 rpm/min, 30 min). The levels of MPO and SOD in the supernatant were determined using the colorimetric method according to the manufacturer's protocol.

### 9 Cell culture and treatment

Murine IEC-6 small intestine follicular epithelial cells cell line, purchased from Cancer Institute of Chinese Academy of Medical Sciences, were grown in Dul-becco's modified Eagle's medium (DMEM, GIBCO, USA) supplemented with 15% FBS (HyClone, Logan, UT), 1% non-essential amino acid, 0.1% sodium pyruvate, and 0.1% bovine insulin. IEC-6 cells were grown in petri dishes at a density of 1×10^6^ cells per well, and randomly divided into 4 groups: control group and LPS group (untreated or treated with 0.1 µl LPS); LPS+curcumin group and LPS+SB positive control group (treated with 10 mmol/L curcumin or 10 mmol/L SB for 30 min before exposure to LPS). At the indicated time points following LPS stimulation, cells were harvested.

### 10 RNA isolation and RT-PCR

Total RNA was isolated using TRIzol reagent (Invitrogen Corporation, Carlsbad, CA) according to the manufacturer's instructions. RT-PCR was carried out using 1 or 3 µg of total RNA from intestinal mucosa from the rat or IEC-6 cells, respectively. The mRNA expression levels of the following 4 genes were quantitated (TNF-α; interleukin-1β, IL-1β; IL-10; ICAM-1). The sequences of PCR primer pairs used for each gene are shown in Tab. 1. GADH and β-actin were used as invariant housekeeping gene internal controls. RT-PCR was performed by annealing at 58°C with 30 cycles.

**Table 1:** Sequence of the amplification primers in the 5′ to 3′ orientation.

		Primer	PCR product (bp)
TNF-α	S	CGTCGTAGCAAACCACCAAG	426
	A	CACAGAGCAATGACTCCAAAG	
IL-1β	S	AATACCACTTGTTGGCTTA	130
	A	TGTGATGTTCCCATTAGAC	
IL-10	S	GGTCTTTAGTTCCTCGTA	306
	A	GCTATGTTGCCTGCTCTT	
ICAM-1	S	AACGACGCTTCTTTTGCTC	433
	A	CTCTGGCGGTAATAGGTGTAA	
GAPDH	S	ACCACAGTCCATGCCATCAC	452
	A	TCCACCACCCTGTTGCTGTA	

### 11 ELISA

The amount of IL-10 in rat blood plasma and IEC-6 cell supernatants were determined in duplicate using ELISA kits (R&D System Europe Ltd., UK) as described by the manufacturer

### 12 Western blotting

Whole protein lysates of intestinal tissues and IEC-6 cells were used. Atotal of 30 mg of protein were used per lane. Primary antibodies were anti-total p38antibody, anti-phosphorylated p38 antibody, anti-phosphorylated ERK 1/2 antibody, anti-phosphorylated JNK 1/2 antibody (both 1∶1000, Cell Signaling Technology, USA), anti-I-κB antibody, anti-MKP-1 antibody, anti-phosphorylated MKP-1 antibody (both 1∶1000, Santa Cruz, USA). Secondary antibodies were horseradish peroxidase-coupled anti-rabbit (1∶5000, Santa Cruz, USA). Chemoluminescence (ECL, Amersham, NO) was used for detection, according to the manufacturer's protocol.

### 13 Immunofluorescence

Nuclear extracts were prepared as described previously [21]. Use NF-κB activation-translocation detection kit to detect the location of NF-κB, according to the manufacturer's instructions.

### 14 Statistical analysis

SPSS11.5 was used as the statistical software. All analyses were showed as mean±standard deviation (SD). Group comparisons were performed using the one-way analysis of variance (ANOVA) test and correlations were tested by Pearson's rank correlation coefficient. p values <0.05 were considered statistically significant.

## Results

### 1 Curcumin possesses anti-inflammatory effect in the MTX-induced enteritis rat models

First, we examined weight, stool viscidity and bloody stools of experimental rats to get DAI scores. Second, we observed mucosa samples of enteritis rats to get CMDI and HS scores (The DAI, CMDI and HS scores were shown in Tab. 2). We found that DAI, CMDI and HS scores of experimental groups treated with curcumin or NAC were lower than those of MTX groups (p = 0.000). But no significant difference between MTX+curcumin group and MTX+NAC group were observed in these indexes (p = 0.65). The results above illustrated that curcumin could obviously alleviate the inflammation reaction and symptoms of enteritis in rats.

**Table 2:** The DAI, CMDI and HS scores in four Groups.

Group	Time (day)	Dose (mg/kg)	DAI	CMDI	HS
Control	6	—	0.12±0.09*	0.30±0.19*	0.16±0.12*
MTX	6	—	2.99±1.02	2.92±1.14	2.84±1.20
MTX+Cur	6	100	1.50±0.87* ^# Δ^	1.81±0.80* ^# Δ^	1.89±1.31* ^# Δ^
MTX+NAC	6	100	1.51±0.60* ^#^	1.59±0.79* ^#^	1.90±0.89* ^#^
F	—	—	56.458	987.235	153.362
P	—	—	0.000	0.000	0.000

* p = 0.000 *vs*. control group;

#p = 0.000 *vs*. MTX group;

ΔP = 0.65 *vs*. MTX+NAC group (n = 15, mean ±SD).

### 2 Curcumin decreases the levels of D-lactate and DAO in the intestinal mucosa

**Figure 2:**
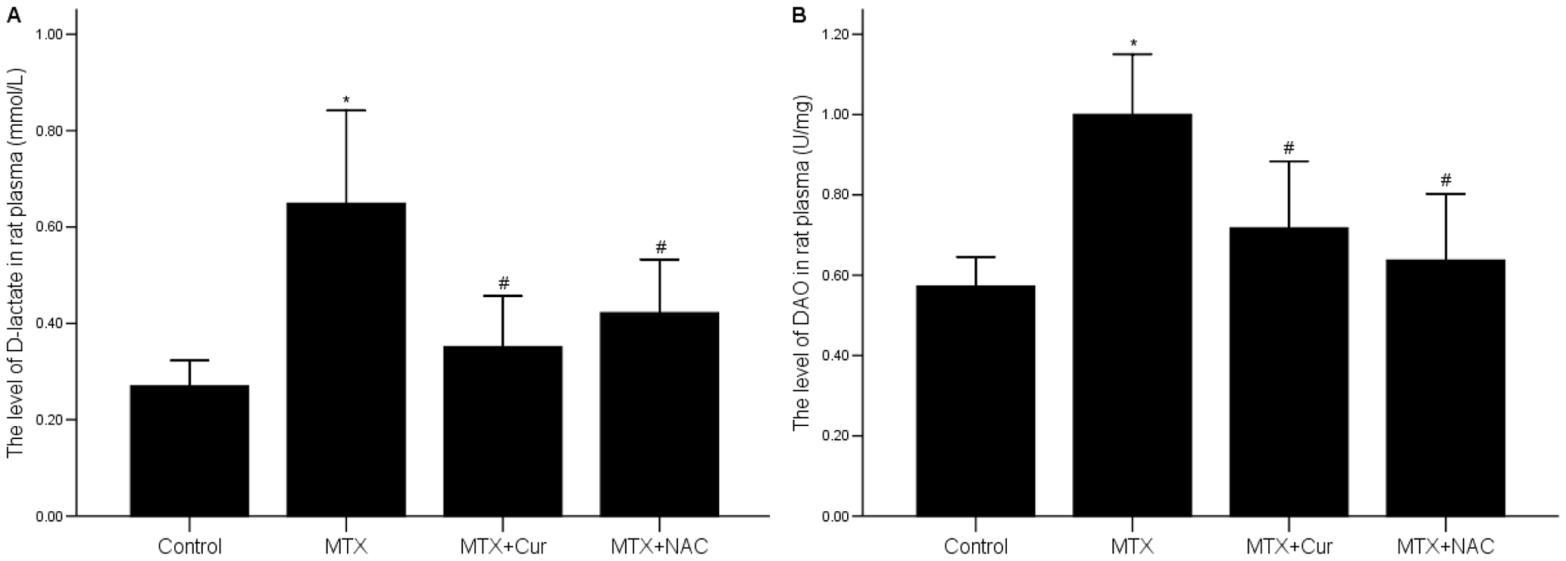
Effects of curcumin on DAO and D-lactate activities in small intestinal mucosa. (A) MTX distinctly increased the level of D-lactate, but curcumin depressed the level of it. (B) The level of DAO were highter in MTX group compared to control group but lower in MTX+curcumin group The same results were found in MTX+NAC group. The levels of DAO and D-lactate of the small intestinal were detected by UV-spectrophotometry. *p = 0.000 vs. control group, #p = 0.000 vs. MTX group.

Spectrophotometry results revealed that the levels of D-lactate and DAO in MTX group were distinctly higher than those in control group (p = 0.000), but the levels of D-lactate and DAO in MTX+curcumin group and MTX+NAC group were markedly lower than those in MTX group in the same period (p = 0.000) (Fig. 2).

### 3 Curcumin decreases the levels of MPO and ICAM-1 in rat enteritis intestinal mucosa and in LPS treated IEC-6 cells

**Figure 3:**
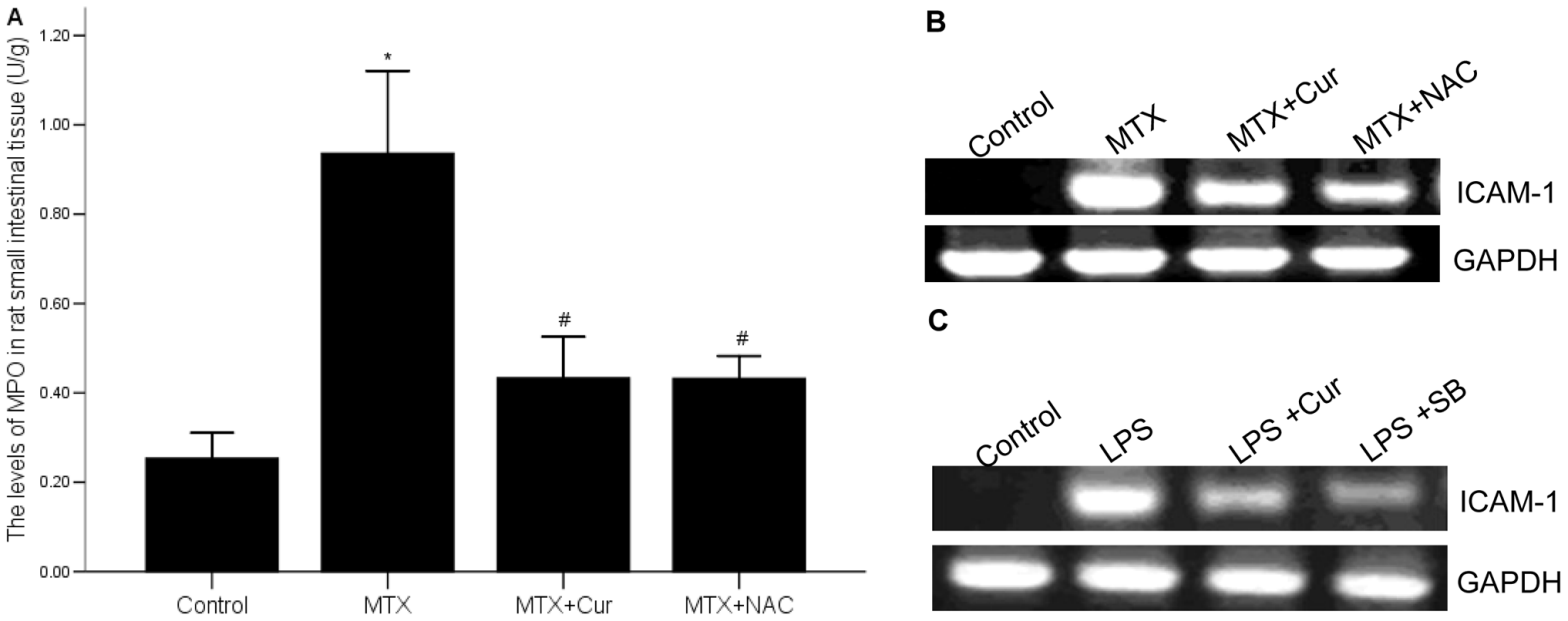
Effects of curcumin on MPO and ICAM-1 activity in vivo and in vitro. (A) The level of MPO in small intestinal mucosa was increased after treated with curcumin or NAC. The level of MPO was determined using the colorimetric method. *p = 0.000 vs. control group, #p = 0.000 vs. MTX group. (B) ICAM-1 mRNA was down-regulated by curcumin or NAC in the intestinal mucosa of experiment rats. (C). ICAM-1 mRNA was down-regulated by curcumin or SB in LPS treated IEC-6 cells. The levels of ICAM-1 mRNA were detected by RT-PCR.

We showed that in MTX group, the level of MPO in rat enteritis intestinal mucosa was significantly higher than it in control group p = 0.000), but in MTX+curcumin group and MTX+NAC group, it was markedly decreased (p = 0.000). We found the similar results in the MTX+NAC group. Furthermore, ICAM-1 mRNA was also down-regulated in MTX induced enteritis rat models and LPS treated IEC-6 cells after treated with curcumin (Fig. 3).

### 4 Curcumin suppresses the expression of pro-inflammatory cytokines in rat enteritis mucosa and supernatant of LPS treated IEC-6 cells

**Figure 4:**
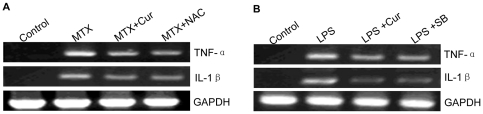
Effects of curcumin on the mRNA levels of TNF-α and IL-1β. (A) The expressions of TNF-α and IL-1β mRNA in MTX group were increased, but it was inhibited after treating with curcumin or NAC. (B) The expressions of TNF-α and IL-1β in LPS treated IEC-6 cells were also increased, but it was depressed by curcumin or SB. The levels of TNF-α and IL-1β mRNA were detected by RT-PCR.

To detect whether curcumin has the same effect on the expression of RT-PCR assays. We demonstrated that expression of TNF-α and IL-1β mRNAs were alldecreased by treating with curcumin or NAC in rat enteritis model (Fig. 4). We also confirmed the same result in LPS treated IEC-6 cells (Fig. 4).

### 5 Curcumin increases the level of IL-10 in rat blood plasma in MTX group and in the supernatant of LPS treated IEC-6 cells

**Figure 5:**
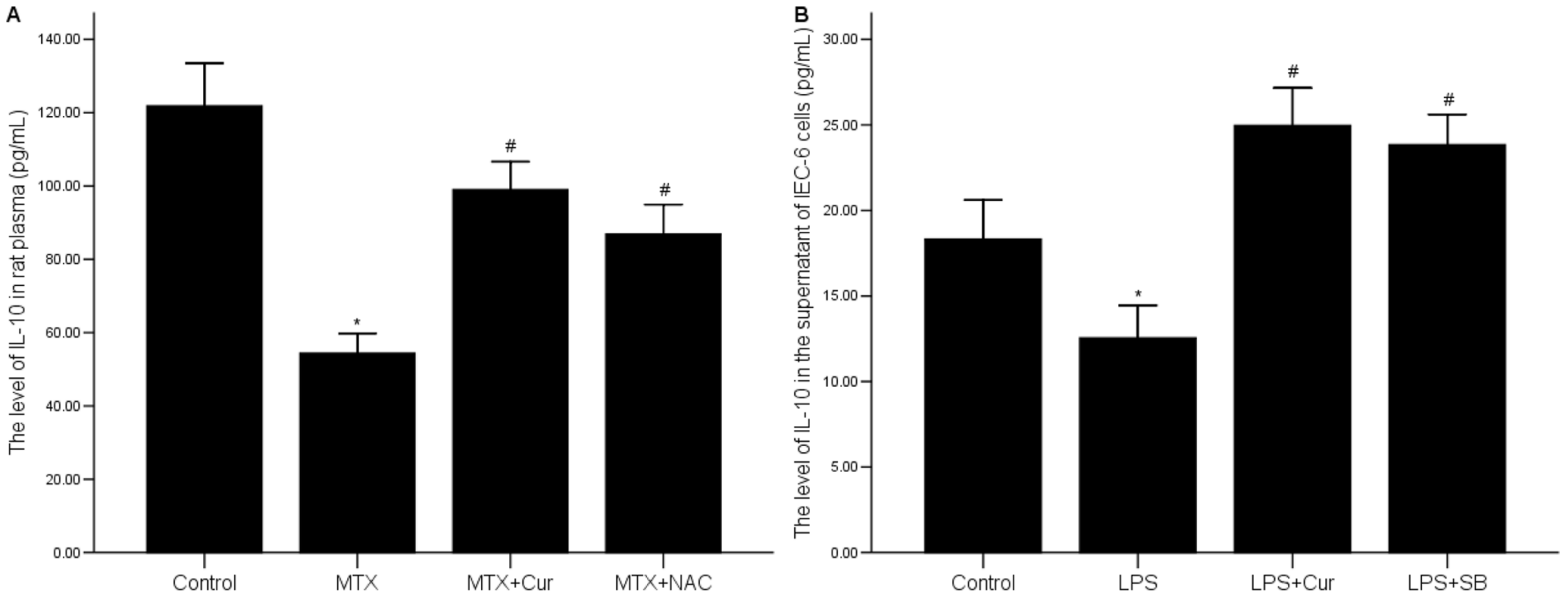
Effects of curcumin on the level of IL-10. (A) The level of IL-10 was decreased in MTX group, but increased after treating with curcumin or NAC. *p = 0.000 vs. control group, #p = 0.000 vs. MTX group. (B) The level of IL-10 was decreased in the supernatant of LPS treated IEC-6 cells. Both Curcumin and SB promoted the expression of IL-10. *p = 0.000 vs. control group, #p = 0.000 vs. LPS group. The level of IL-10 was detected by ELISA.

The expression level of IL-10, detected by ELISA, was significantly lower in MTX group than it in control group (p = 0.000) (Fig. 5). The expression level of IL-10 in MTX+curcumin group and MTX+NAC group were higher than it in MTX group (p = 0.000). We found no significant differences of IL-10 expression between the MTX+curcumin group and MTX+NAC group (p = 0.43). We showed the same result in LPS treated IEC-6 cells (Fig. 5)

### 6 Curcumin induces the level of SOD in the intestinal mucosa

**Figure 6:**
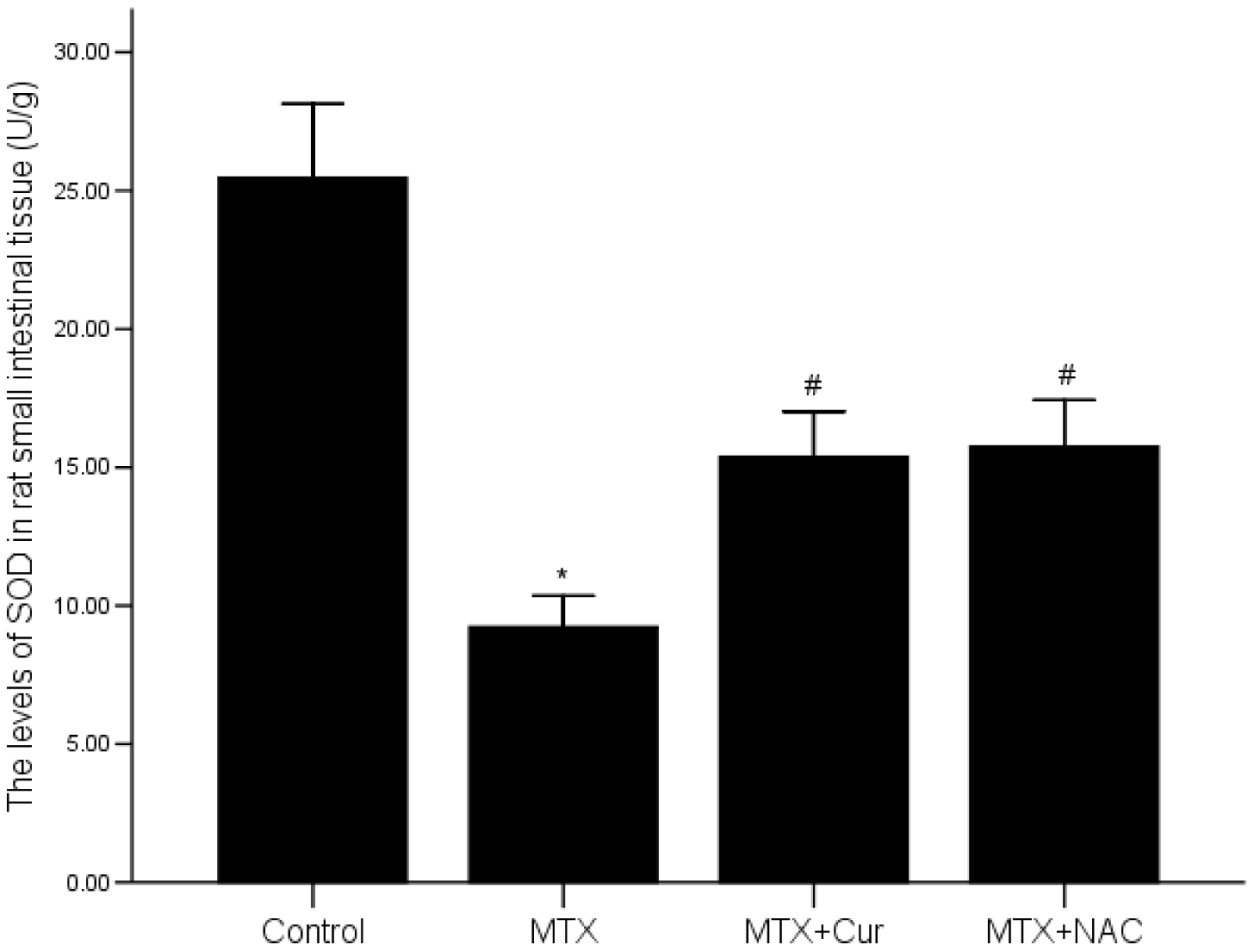
Effects of curcumin on small intestinal SOD activity. The level of SOD was inhibited by MTX, while elevated by treating with curcumin or NAC. The levels of SOD in the intestinal mucosa were determined using the colorimetric method. *p = 0.000 vs. control group, #p = 0.000 vs. MTX group.

Our results indicated that the level of SOD was decreased in MTX group compared with control group (p = 0.000), but curcumin or NAC could reverse this effect. The level of SOD was significantly higher in MTX+curcumin or MTX+NAC group than it in MTX group (p = 0.000) but lower than it in control group (Fig. 6).

### 7 Curcumin attenuates phospho-p38 but has no effect on phospho-JNK1/2 and phospho-Erk1/2

**Figure 7:**
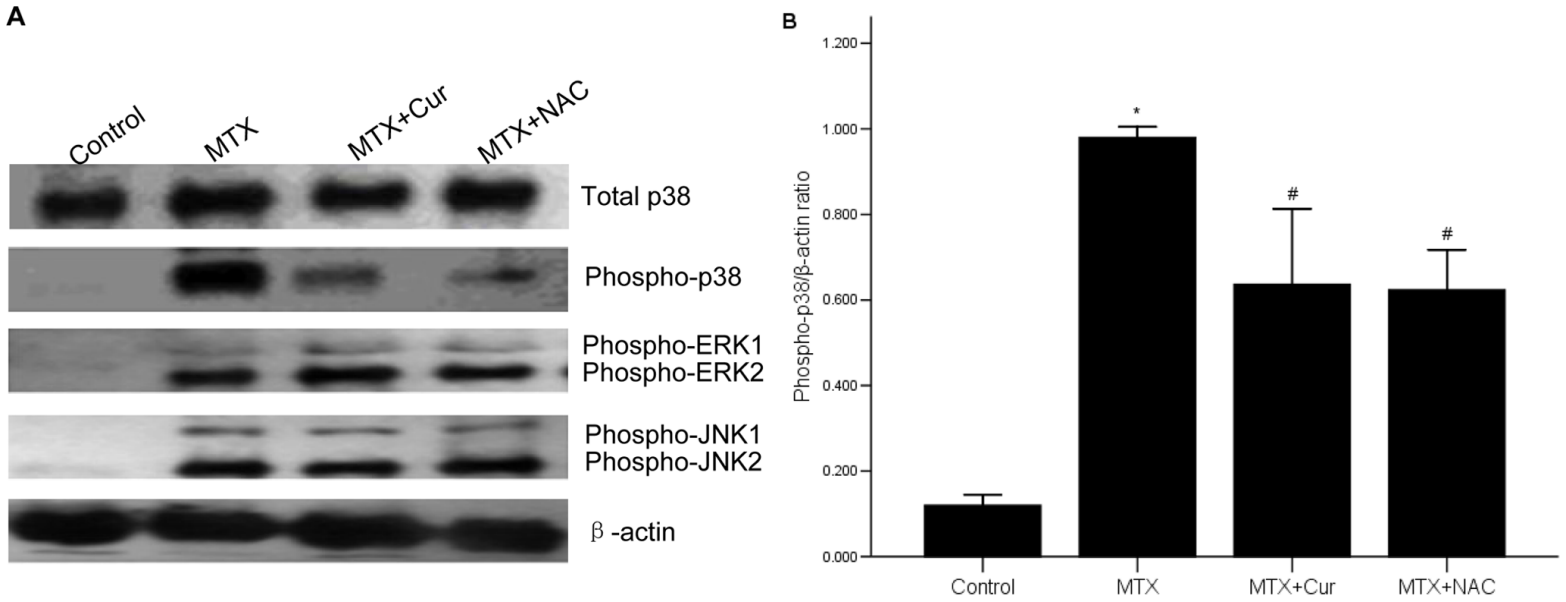
Effect of curcumin on phosphorylation sate of p38. (A) The expressions of total p38, phosphorylation sates of p38, Erk1/2, and JNK1/2 were all enhanced in MTX group. Only the phosphorylation sate of p38 was attenuated by treating with curcumin or NAC. The expressions of phosphorylated or total proteins were detected by western blot. (B) The bar graph was a clear reflection of the level of phosphorylation sate of p38 among four groups. *p = 0.000 vs. control group, #p = 0.000 vs. MTX group.

It is known that inflammation is triggered by intracellular signaling pathway events, which involve the activation of MAPK. In order to further understand the underlying mechanisms of curcumin-mediated anti-inflammation, we examined whether curcumin could inhibit activation of MAPK signaling molecules including p38, Erk1/2, and JNK1/2. We used western bolt to detect the levels of total and phosphorylated p38, phosphorylated Erk1/2, and phosphorylated JNK1/2 related in MAPK signal pathway in the small intestinal tissue of experiment rats following treatment with or without curcumin. Results indicated that the total protein levels of p38 were not changed in four groups. But the phosphorylation sate of p38, Erk1/2, and JNK1/2 were all activated in rat enteritis mucous of MTX group, then we found that treatment with curcumin or NAC resulted in a reduction in p38 phosphorylation (p = 0.000), but not of that in Erk1/2 and JNK1/2 (Fig. 7). These observations suggested that curcumin might inhibit the activation of the p38 kinases but not the Erk1/2 and JNK1/2.

### 8 Curcumin increases the expression of phospho-MKP-1 in LPS-stimulated IEC-6 cells

**Figure 8:**
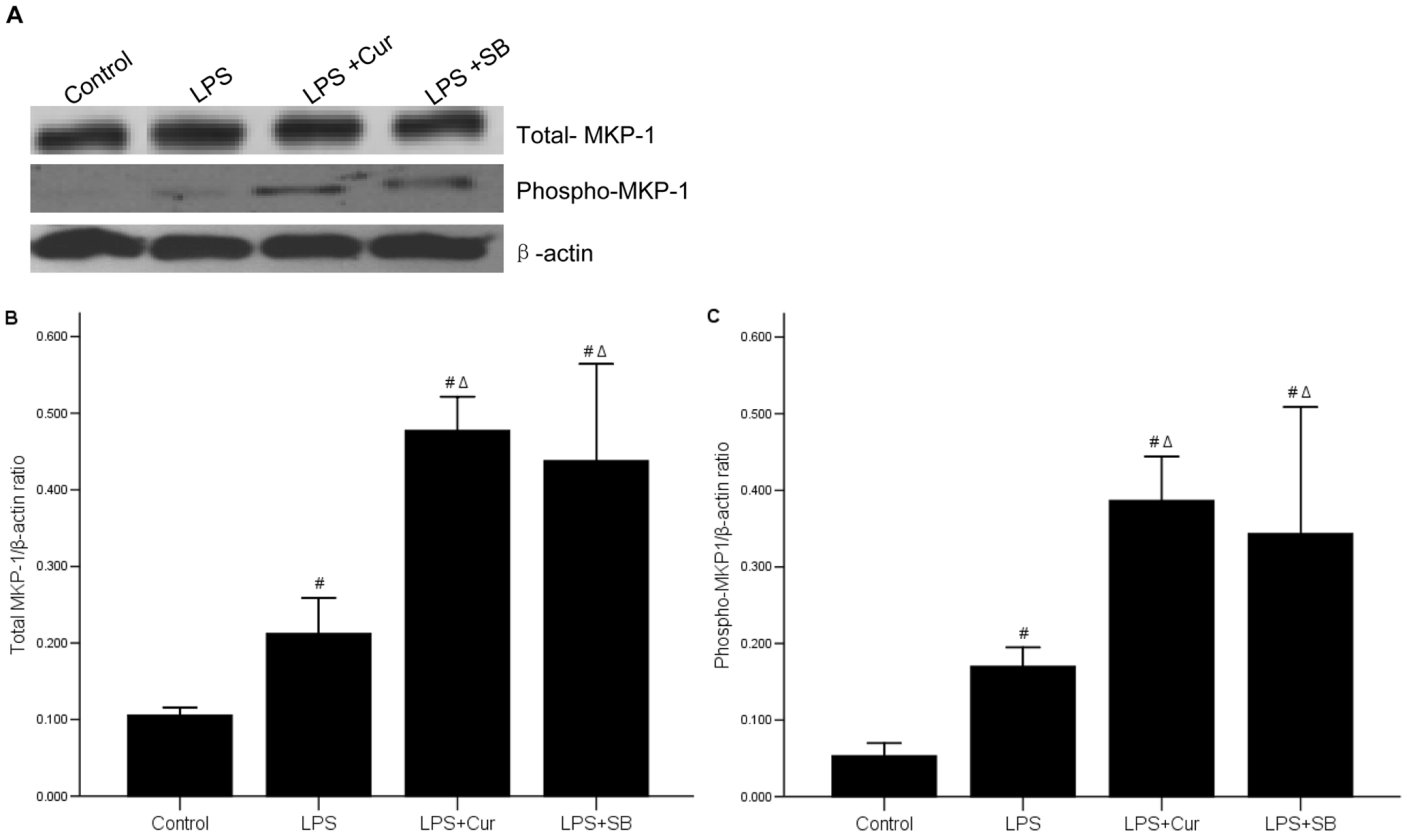
Effect of curcumin on both total MKP-1 and its phosphorylation state in LPS-stimulated IEC-6 cells. (A) The expressions of total MKP-1 and phospho MKP-1 were highest in LPS+curcumin group and LPS+SB group, middle in LPS treated IEC-6 cells, and lowest in control group. The levels of total or phosphorylated proteins were detected by western blot. (B) (C) The bar graphs were a clear reflection of the level of total MKP-1 and phosphor-MKP-1 among four groups. #p<0.05 vs. control group; Δp = 0.00 vs. LPS group.

MKP-1, one of the dephosphorylated factors of MAPK, which can inhibit the activity of ERK, JNK and p38 in stress reaction[22], [23], participates in regulation of multiple physiological and pathological processes, including inflammatory reaction by regulating the activity of ERK, JNK and p38. Recent findings showed that MKP-1 deficiency could induce the activation of p38 and JNK phosphorylation. This prompted us to explore whether MKP-1 was involved in the curcumin-induced attenuation of p38. We examined total MKP-1 and its phosphorylation state in LPS-stimulated IEC-6 cells (Fig. 8). Results indicated that LPS alone enhanced both total and phosphorylated MKP-1 compared with those in control group (p<0.05), and that curcumin or SB in combination with LPS further enhanced the levels of MKP-1 phosphorylation, compared to those observed in the presence of LPS alone (p = 0.000). This demonstrated that LPS could increase the expression of total and phospho-MKP-1, while curcumin or SB might amplify this result.

### 9 Curcumin inhibits LPS-induced degradation of I-κB and restrains translocation of NF-κB and pro-inflammatory cytokine product

**Figure 9:**
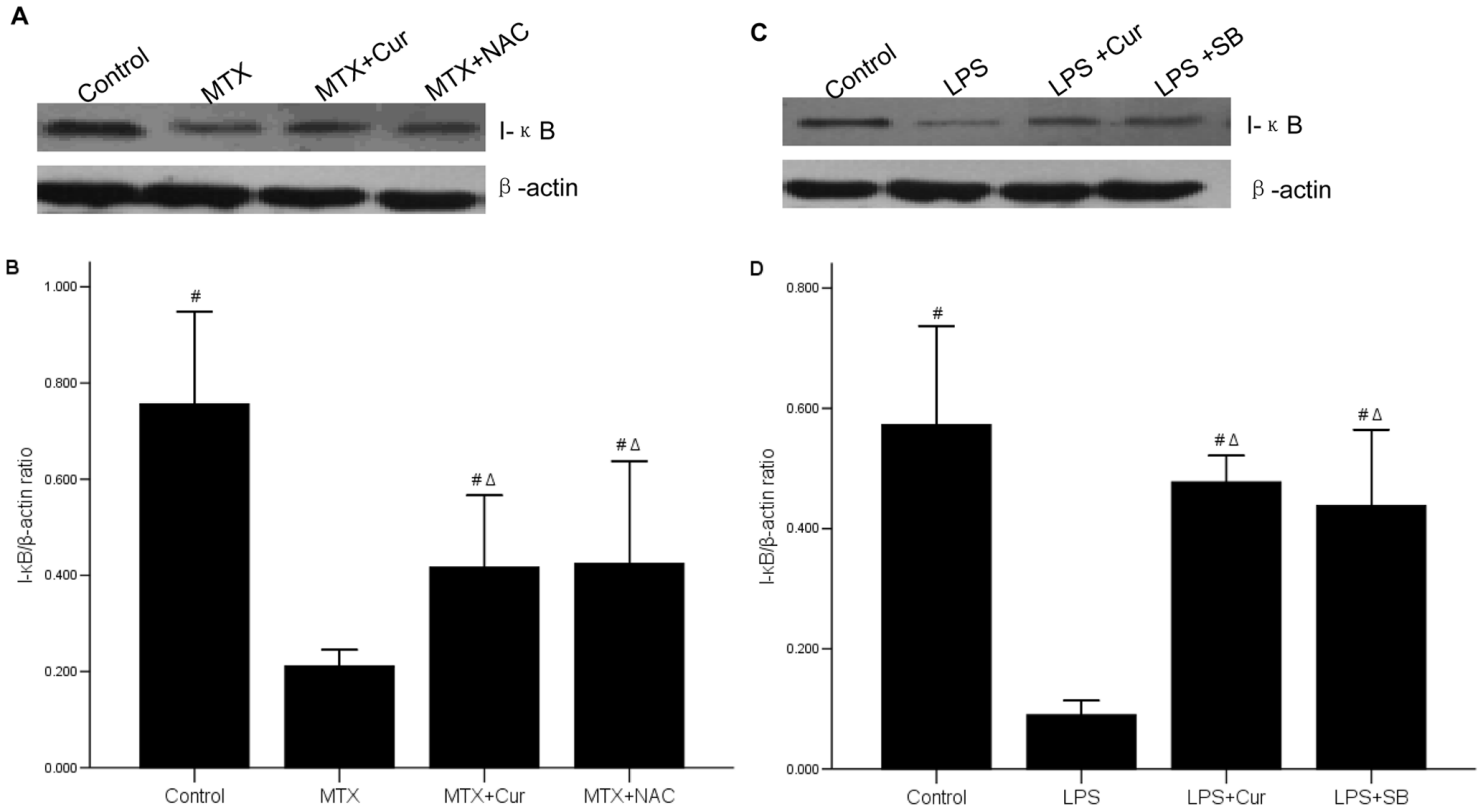
Effect of Curcumin on inhibition of I-κB degradation. (A) The level of I-κB protein in the small intestinal tissue was decreased in MTX group, but increased by curcumin or NAC. (B) The bar graph was a clear reflection of the level of I-κB among four groups. #p = 0.000 vs. MTX group, Δp<0.01 vs. control group (C) The expression of I-κB protein was decreased in LPS group, but increased after treating curcumin or SB. (D) The expression of I-κB protein in LEC-6 cells was showed in bar graph. #p = 0.000 vs. LPS group, Δp<0.01 vs. control group. The level of I-κB protein was detected by western blot.

Beside MAPK signal pathway, NF-κB pathway is the most important downstream signal transduction pathway mediated by LPS [24]–[27]. Some studies have shown that activation of MAPK phosphorylation transduction signals could activate downstream transcription of NF-κB-mediated pro-inflammatory cytokines. We intended to investigate whether curcumin could result in inactivation of NF-κB signal pathway. First, we evaluated the protein level of I-κB to which inactivated NF-κB was bounded. We found that the expression of I-κB in control group was higher than it in MTX group (p = 0.000), while it in MTX+curcumin group and MTX+NAC group were both lower than it in control group (p<0.01) but higher than it MTX group (p = 0.000) (Fig. 9). Curcumin played the same role as SB in LPS treated IEC-6 cell (Fig. 9).

**Figure 10:**
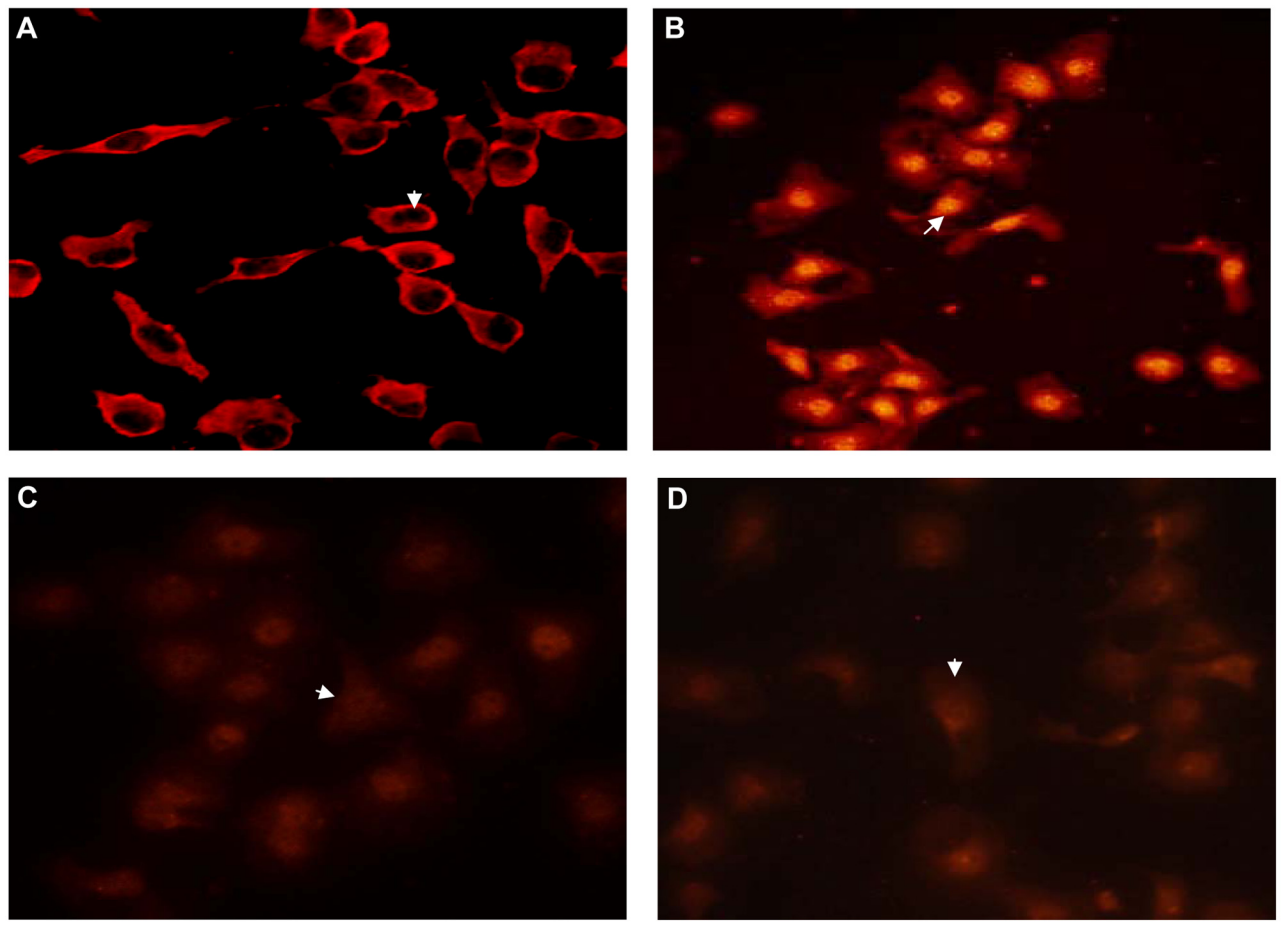
Effect of curcumin on blocking NF-κB translocation into nucleus. (A) NF-κB p65 was located in the cytoplasm in control group (white arrow). (B) NF-κB p65 translocated to nucleus after stimulated by LPS (white arrow). (C) Curcumin attenuated the translocation of NF-κB p65 to nucleus (white arrow). (D) SB inhibited the translocation of NF-κB p65 into the nucleus (white arrow). These were observed by immunofluorescence. Magnification×400.

Then, we examined whether curcumin could block NF-κB translocation into nucleus, since nuclear translocation was often recognized as cell reaction to LPS stimulation and seemed to correlate with NF-κB-mediated transcriptional activation. So, we measured the level of NF-κB p65 in the nucleus of LPS-stimulated IEC-6 cells in the presence or absence of curcumin in order to indentify whether NF-κB were activated. We clearly showed that NF-κB p65 expressed in endochylema but not in nucleus in normal IEC-6 cells (Fig. 10). LPS stimulation led to an increase in p65 levels in the nucleus, while treatment of 1.curcumin or SB restrained this effect (Fig. 10). According to this, we suggested that curcumin not only indirectly suppressed activation of NF-κB but controlled the translocation of NF-κB into the nucleus *in vitro*. This was the same as the inhibitory effect of SB.

## Discussion

Intestinal epithelial barrier, including biotic barrier, mechanical barrier and immunity barrier, is known as a very crucial barrier against pathogen infection,especially the immunity barrier. Up to now, there is no effective way to prvent IMB damage. It is necessary to develop natural medicines effective for IMB dysfunction and the intercurrent diseases.

In this report, we focused on curcumin with anti-inflammation and anti-oxidization effects and commonly used in traditional medicine in many Asian countries. First, in order to indentify the effective role of curcumin *in vivo*, we constructed enteritis animal model by MTX, and detected the two indicators, D-lactate and DAO, to examin extent of damage and repair of rat intestinal tract which associate with IMB function. We found that the general condition, diarrhea and bloody stool symptoms were relieved in MTX+curcumin group and MTX+NAC group. DAI, CMDI and HS were also relieved after treated with curcumin or NAC (Tab. 2). Data showed that curcumin reduced the levels of D-lactate and DAO in MTX-induced rat enteritis model (Fig. 2). This proved that curcumin improved the permeability of the intestinal mucosa, reduced the extent of intestinal mucosa damage and finally protected IMB function.

The pathophysiological process of intestinal structural damage is as follows: intestinal mucosa cells release chemokine and cytokines after stimulated by causative factors; inflammatory cells infiltrate and release inflammatory mediators; finally lead to cell death or apoptosis [28]. Leukocyte's over-activation is a very important knot of this process. The level of MPO, an enzyme mainly existing in neutrophil, reflects the level of neutrophil infiltration. We indicated that the level of MPO was clearly decreased in curcumin or NAC treated group (Fig. 3A), which suggested curcumin inhibited neutrophil infiltration *in vivo*. In addition, Leukocyte's stagnation and infiltration also depend on the expression and function of ICAM, one kind of glycoprotein, which promotes cell-cell or cell extracellular matrix adhesion. Moreover, ICAM-1, belonging to the immunoglobulin superfamily, has received the attention in recent years in the process of IBD[29]–[30]. Our data showed that the mRNA expression of ICAM-1 was significantly suppressed by curcumin *in vitro* and *in vivo* (Fig. 3B and C). All of this results suggested that curcumin played anti-inflammatory role partly by inhibiting neutrophil infiltration and its over-activation.

The imbalance of pro-inflammatory cytokines and anti-inflammatory cytokines is another important injury mechanism of intestinal mucosal. IL-1β, a classic pro-inflammatory cytokine, is an important mediator of inflammation in variety of clinically stressful conditions. It also served as the mediator for intestinal mucosa injury[31]–[33]. TNF-α participates in early steps of inflammation, causes aggregation of inflammatory cells and plays important roles in inducting expression of other inflammatory cytokines[34]. In the pathogenesis of IBD, TNF-α participates in the progress of granuloma formation. To study the effect of curcumin on pro-inflammatory cytokines, we used RT-PCR to determine the mRNA level of IL-1 and TNF-α. Our results showed that the mRNA expressions of IL-1 and TNF-α were significantly suppressed by curcumin in intestinal mucosa of MTX induced rat models and LPS-induced IEC cells (Fig. 4). On the other hand, IL-10, identified as an anti-inflammatory cytokine, suppresses T lymphocytes and mononuclear cell function and many pro-inflammatory cytokines [35]–[37]. We detected the level of IL-10 by ELISA, and discovered that it was up-regulated by curcumin *in vivo* and *in vitro* (Fig. 5A and B). Curcumin down-regulated pro-inflammatory cytokine expression, while up-regulated anti-inflammatory cytokine production in *vivo* and *in vitro*, thereby, the anti-inflammatory effect of curcumin was further confirmed.

Additionally, reactive oxygen species (ROS) plays a “trigger” role in the pathophysiological process of intestinal structural damage [38]–[40]. We tested curcumin for its ability to inhibit the combined inflammatory and oxidative damage which occured as a response to inflammatory in the enteritis rat models. Researchers have proved that anti-oxidant function of intestinal mucosa was damaged in the animal model of salmonella infection, chronic diarrhea and ulcerative colitis, and oxygen free radical scavenger could be used to treat such diseases [41]–[43]. The anti-oxidant defense system in the intestinal mucosa for eliminating ROS, contains enzymes system and non-enzymes system. The former includes SOD, Catalase (CAT), and Glutathione peroxidase (GSH-Px). Here, we selected SOD, the endogenous superoxide anion radical scavenger, as an anti-oxidant indicator, because SOD showed the body's ability of eliminating free radicals. Our results demonstrated that the level of SOD was increased in the MTX induced rat intestinal mucosal after treated with curcumin (Fig. 6). Based on this, we suggested that curcumin has certain effect on anti-oxidant and eliminating free radicals.

Next, we made in-depth research on possible molecular mechanisms of curcumin. Accumulating evidence supported that intestinal injury, including ischemic, inflammation, apoptosis and other pathological mechanisms were related with the regulation of MAPK [20] and NF-κB signal pathway [15], [21], [44]–[50]. The expression of IL-10 is under the control of the Sp1 transcription factor that is also regulated by MAPK pathway [51]. Therefore, we disscused the role of curcumin on two pathways. MAPK which consists of three major subgroups, ERK1/2, JNK1/2 and p38 MAPK plays a key role in transducing various extracellular signals to nucleus and regulating cell growth and differentiation. Moreover, MAPK takes part in the LPS-mediated signal transduction pathway [52]–[54] and controls cellular responses to cytokines and stressors. In present study, we demonstrated that curcumin restrained the phosphorylation of p38 MAPK, but not ERK1/2 and JNK1/2 (Fig. 7A and B). These suggested that the anti-inflammatory effect of curcumin was partly due to the inhibition of p38 activity.

MKP-1, another special MAPK family, is capable of dephosphorylating and inactivating various members of the MAPK family [55], [56]. It is reported that MKP-1 deficiency enhanced phosphorylation of p38 and JNK [57]. Our results revealed that both total MKP-1 and phospho-MPK-1 was obviously activated by treatment with curcumin in LPS treated IEC-6 cells (Fig. 9). Thus we proved that curcumin might inhibit the phosphorylation of p38 by activating MKP-1, and eventually reduced the inflammatory response. Curcumin had no effect on the ERK1/2 and JNK1/2 MAKP might be because these were not effective downstream substrate of MKP-1.

Besides, NF-κB is known as a key factor in up-regulating inflammatory cytokines. NF-κB activation enhances the transcription of pro-inflammatory cytokines, and the cytokines are known to in-turn activate NF-κB [58]. Thus we still detected whether curcumin could regulat NF-κB signal pathway. NF-κB is located in cytoplasm and bounds to I-κB as an inactive complex. The phosphorylation and subsequent degradation of I-κB result in separation of the complex, and then NF-κB is activated. The activated NF-κB migrates into the nucleus, and causes the expression of inflammatory cytokines, such as TNF-α, IL-6 and IL-8 [59]. Meanwhile, NF-κB can be activated by inflammatory factors such as IL-1β and TNF-α [60]. In addition NF-κB signaling pathway is the downstream pathway of LPS-mediated transduction pathways. Here, we displayed the level of I-κB was degradated in MTX-induced rat intestinal mucosa and LPS-treated IEC-6 cells, while it was increased after given curcumin (Fig. 9). Hence, we considered that the degradation of I-κB was abolished when treated with curcumin *in vivo* and *in vitro*. These observations explained that curcumin could inhibit I-κB degradation, while indirectly repressed NF-κB activation. Then we further detected the effect of curcumin on NF-κB translocation. Immunofluorescence data showed that LPS promoted translocation of NF-κB p65 into nucleus, but curcumin inhibited this effect (Fig. 10). The above-mentioned data showed that curcumin could not only inhibit the activation of NF-κB but also control the translocation of NF-κB. We suggested the inhibitory effects of curcumin on the production of inflammatory factors and cytokine probably occurred via the NF-κB signaling pathway.

Overall, our results proved that curcumin has effect on anti-inflammation, anti-oxidation and free radical removal not only *in vitro* but *in vivo*. We futher demonstrated curcumin restrained the activation of p38 MAPK via enhancing MKP-1 phosphorylation, but not ERK1/2 and JNK1/2 MAPK *in vitro*. Besides, our results suggested that curcumin restrained the activation and translocation of NF-κB. Taken together, we have a better understanding of the molecular mechanism of curcumin on protecting IMB. Such effect is mediated by a blocking of p38 MAPK via enhancing MKP-1 phosphorylation and inhibiting NF-κB activation. We suggested curcumin had a remarkable protective effect for IMB and therapeutical effect on various human inflammatory diseases.
